# Large local reaction to Hymenoptera stings: Sound studies are needed to change a shared concept

**DOI:** 10.1002/iid3.268

**Published:** 2019-09-07

**Authors:** Stefano Pucci, Cristoforo Incorvaia, Antonino Romano

**Affiliations:** ^1^ General Hospital Civitanova Marche Allergy Unit Civitanova Marche Italy; ^2^ ASST Pini‐CTO Cardiac/Pulmonary Rehabilitation Milan Italy; ^3^ Allergy Unit Presidio Columbus Rome Italy

**Keywords:** culprit insects, Hymenoptera venom allergy, large local reactions, natural history, systemic reactions

## Abstract

The natural history of large local reactions to Hymenoptera stings allowed to estimate the risk to develop a systemic reaction after an initial large local reaction in about 4% of patients. A recently published study claimed that such risk concerns instead around one‐fourth of patients. However, such study is flawed by serious imprecision, particularly the unreliable identification by patients of the culprit insect, as well as the dubious identification of the causative venom in multisensitized patients. Also, the authors criticized previous studied because of the limited number of patients, while they included in the study 662 patients. Indeed, when only patients clearly restung by the same insect according to their history data were considered, the number of patients fell to 35. These data are unable to change the current shared concept on the low risk of systemic reactions in patients with initial large local reaction.

Allergic reactions to Hymenoptera venom include systemic sting reactions (SSR), which when clinically expressed as anaphylaxis are burdened with mortality,^1^, ^2^ and large local reactions (LLR). These are defined by skin reactions around the site of the sting characterized by edema, erythema, and itching, with a diameter greater than 10 cm.^3^ The risk to develop a SSR from the same Hymenoptera species after an initial LLR is generally considered as low both in adults and children.^4^, ^5^ In particular, the rate of SSR of about 4% reported by Mauriello in 1984^6^ in patients with a single LLR was recently confirmed.^7^ Sturm et al^8^ have expanded the analysis of the issue. The study had as primary aim to evaluate the relevance of asymptomatic sensitizations to insect venom by sting challenges, but it was observed indeed that 7.4% of 25 subjects with a previous LLR reacted systemically to the sting challenge and that 4.5% of 64 subjects had SSR without a previous LLR, this difference being not significant. In addition, resting challenges were performed in 18 subjects. Around 44% had LLR after the first sting challenge and again no SSR occurred after the second challenge.

In a recent article, Bilò et al^9^ claim to change the concept of the low risk of systemic reactions in patients with LLR to Hymenoptera stings, based on their observation that in a population of 662 patients with LLR about a quarter developed an SSR to subsequent stings. They also found that a higher risk of subsequent SSR was associated with a skin test reactivity to *Apis mellifera* or *Vespula species* at 0.001 µg/mL concentration (odds ratio, 13.4 and 16.5, respectively). This would be interesting if the study was not seriously flawed. First, it is known that the general population is not skilled in identifying stinging insects with the exception of the honeybee^10^ (especially concerning beekeepers). In fact, in table 1 the insects are defined as “presumed.” Did the authors verify the skilfulness in the 350 patients who recognized vespids? Moreover, the majority of patients were multisensitized, and in such patients, the diagnostic agreement between patient's description of the stinging insect and the result of skin test was much lower (*k* = 0.50 for honeybee and 0.60 for vespids) than in monosensitized patients for distinguishing honeybee from vespids (*k* = 0.95). Also, it is not reported if patients were enabled to recognize the culprit insects in subsequent stings. As to study population, the authors criticize previous studies for “small patient sample size,” but in the Results section, it is written “Among the 35 patients clearly re‐stung by the same insect—according to their history …”. Thus, based on the number of certain identifications, also Bilò et al used a small sample. Of note, when calculated in these patients, the rate of systemic reactions was 11%, much lower than the 24% in the entire population which included patients not clearly restung by the same insect. In conclusion, the absence of essential data make the results of the study questionable. In the Discussion section, the authors mentioned the possible role of sting challenges and explained that they did not use it because “is not recommended for diagnostic use, due to several related issues (ie, difficult choice of the right insect to test—based on uncertain clinical history and diagnostic test outcome.” Unfortunately, the same uncertainty concerns the study by Bilò et al. However, there is no reason to doubt the good intentions of the authors. Given that a large number of allergy centers took part in the study, it is possible to expect a new study to be planned that includes only patients who have certainly recognized the culprit insect, with confirmation of a positive result from skin tests or in vitro tests for that insect.
